# Nose–brain axis: A bridge from the nasal cavity to the central nervous system

**DOI:** 10.4103/NRR.NRR-D-25-01770

**Published:** 2025-12-31

**Authors:** Guohui Yang, Dongdong Zhu, Kaizhi Zhang

**Affiliations:** 1 Department of Otolaryngology–Head and Neck Surgery, China–Japan Union Hospital of Jilin University, Changchun, Jilin Province, China; 2 Department of Neurosurgery, China–Japan Union Hospital of Jilin University, Changchun, Jilin Province, China

**Keywords:** Alzheimer’s disease, blood‒brain barrier, central nervous system diseases, intranasal drug delivery, nasal inflammation, neuroimmune interaction, nose‒brain axis, Parkinson’s disease

## Abstract

The nose–brain axis is a direct pathway linking the nasal cavity to the central nervous system. Odors, as well as exogenous substances such as pathogens, inflammatory mediators, and drugs, can enter the cranial cavity through pathways including the olfactory nerve, trigeminal nerve, and humoral routes, thereby enabling signal transmission and material exchange from the peripheral nasal cavity to the central nervous system. In recent years, advances in multimodal visualization technologies have made it possible to dynamically monitor the nose–brain axis from the molecular level to the tissue level, providing important means for revealing its functional characteristics and pathological changes. Owing to the existence of the nose–brain axis, nasal inflammation can, through neuro-immune interactions, activate central microglia and astrocytes and induce neuroinflammation, thus promoting the onset and progression of central nervous system diseases. In addition, the nose–brain axis offers a unique route for the treatment of central nervous system disorders. Intranasal drug delivery can bypass the blood–brain barrier, act directly on the central nervous system, increase intracranial drug bioavailability, and produce rapid effects, providing new ideas for treating cross-system diseases. This review systematically summarizes the anatomical pathways of the nose–brain axis, visualization monitoring technologies, and mechanisms by which nasal inflammation affects the central nervous system. It also reviews advances in intranasal drug delivery for emotional disorders, migraine, Parkinson’s disease, and Alzheimer’s disease, aiming to provide new strategies for studying the mechanisms by which nasal inflammation influences the central nervous system and for cross-system targeted therapy.

**Facts**

• The nose‒brain axis (NBA) constitutes a direct, multi-pathway communication network linking the nasal cavity to the central nervous system (CNS), enabling both neural and humoral transmission of signals and substances.

• Nasal inflammation can trigger CNS neuroinflammation via the NBA by activating microglia and astrocytes, leading to elevated cytokine levels and contributing to the pathogenesis of disorders such as depression, migraine, Parkinson’s disease, and Alzheimer’s disease.

• Intranasal drug delivery via the NBA can bypass the blood‒brain barrier, significantly increasing CNS bioavailability and enabling rapid, targeted therapeutic effects in neurological and psychiatric conditions.

**Open questions**

• How can intranasal drug delivery be optimized to improve reproducibility and targeting efficiency across individuals with diverse nasal anatomies and mucosal conditions?

• What are the long-term safety and neurotoxicity profiles of repeated intranasal administration, particularly when nanoparticle carriers or biologics are used for chronic CNS diseases?

• To what extent does chronic nasal inflammation contribute to the initiation or progression of neurodegenerative diseases such as Parkinson’s disease or Alzheimer’s disease, and can early intervention via the NBA alter disease trajectories?

## Introduction

Traditionally, the nasal cavity has been regarded as the starting point of respiration and olfaction, with its functions believed to be limited to air filtration and odor perception. However, increasing evidence in recent years has demonstrated a direct structural and functional connection between the nasal cavity and the central nervous system (CNS), known as the nose–brain axis (NBA). NBA not only plays a role in olfactory transmission and odor perception but also has important significance in neuroimmune regulation, disease propagation, and drug delivery (Mou et al., 2024b).

The NBA operates primarily through three parallel and potentially cooperative pathways: (1) Olfactory nerve pathway: The olfactory nerve, the only CNS nerve fiber exposed to the external environment, conveys external signals from the olfactory mucosa directly to the olfactory bulb and then to related brain regions through tertiary projections (Zhou et al., 2019). (2) Trigeminal nerve pathway: The trigeminal nerve contains abundant nerve endings in the nasal mucosa, transmitting signals through the trigeminal ganglion to the brainstem and subsequently to other brain regions (Hirata et al., 2004). (3) Humoral pathway: In recent years, the existence of an intracranial lymphatic system has been proposed. This system communicates with the nasal lymphatic system and the circulatory system, enabling the exchange of peripheral and central fluids (Chae et al., 2024). Together, these pathways constitute a bidirectional network that not only enables sensory transmission but also allows odor molecules, pathogens, inflammatory mediators, and drugs to bypass the blood–brain barrier (BBB) and be directly transported between the nasal cavity and the brain. With advances in visualization and monitoring technologies—from early histological tracing to the latest multimodal *in vivo* imaging techniques—researchers are now able to dynamically and noninvasively monitor NBA from the molecular to the tissue level, providing technical support for clarifying its functional characteristics and alterations under different physiological and pathological conditions.

A growing body of research indicates that nasal inflammation plays a key role in the onset and progression of CNS diseases. Following bacterial infection or allergen exposure, the nasal mucosa releases large quantities of cytokines that can travel along the olfactory and trigeminal pathways into the CNS, accompanied by imbalances in neuropeptides and neurotransmitters as well as immune cell infiltration. This neuroimmune interaction is considered a common pathological mechanism linking peripheral inflammation to various CNS disorders, ultimately contributing to emotional disorders, migraine, Parkinson’s disease (PD), and Alzheimer’s disease (AD) (Spekker et al., 2021; Zhou et al., 2023; Zhang et al., 2025). Similarly, increasing evidence indicates that NBA provides a direct route for intranasal drug delivery for CNS disorders. This pathway enables drugs to bypass the BBB, enter the CNS rapidly and directly, increase intracranial bioavailability, and reduce systemic side effects.

Therefore, this review systematically summarizes the anatomical pathways of NBA and advances in visualization technologies; explores in depth how nasal inflammation affects the CNS through neuroimmune interactions; and comprehensively reviews recent applications and prospects of NBA-based intranasal drug delivery strategies in emotional disorders, migraine, PD, and AD. The aim is to provide theoretical foundations and research directions for understanding the pathogenesis of cross-system diseases and for developing novel targeted therapeutic approaches.

## Search Strategy

We adopted a narrative review approach to search and screen the relevant literature. Searches were conducted primarily through the electronic databases PubMed and Web of Science. The keyword combinations included “nose–brain axis,” “olfactory pathway,” “trigeminal nerve,” “humoral pathway,” “nasal lymphatic–cerebrospinal fluid (CSF) clearance,” “visualization techniques,” “nasal inflammation,” “neuroinflammation,” “neuropeptides,” “neural regeneration,” “intranasal administration,” “stem cells,” “exosomes,” “nanoparticle delivery,” “brain targeting,” “neurotrophic factors,” and “neurotoxicity” combined with terms related to “CNS diseases,” “affective disorders,” “migraine,” “PD” and “AD.” The time frame focused on representative cutting-edge studies published within the past 15 years, as well as seminal works outside this period. The included studies directly addressed the anatomical pathways of the NBA, its physiological or pathological mechanisms, its associations with CNS diseases, or intranasal intervention strategies. Eligible study types included original research, reviews, and meta-analyses; subjects included animals or humans; and publications were restricted to the English language. Additionally, the reference lists of the selected articles were examined to identify further important sources. During the analysis, particular attention was given to evaluating the scientific rigor, representativeness, and logical coherence of the study designs, with clear distinctions between clinical research and animal studies.

## Anatomical Pathways of the Nose–Brain Axis and Advances in Visualization Research

After entering the nasal cavity, odorants pass through the turbinates and come into maximal contact with the olfactory sensory neurons located on the dorsal surface of the turbinates. They subsequently bind to olfactory receptors on the dendrites of these neurons, inducing conformational changes in the receptors and generating action potentials within the neurons. These signals are transmitted through the olfactory nerve bundles across the cribriform plate to the ipsilateral olfactory bulb. From the olfactory bulb, projections are sent to the anterior olfactory nucleus and then to the contralateral olfactory bulb, while ipsilateral projections extend to the primary olfactory cortex (including the amygdala, hippocampus, and orbitofrontal cortex), where they form a complex olfactory cognitive network (Soudry et al., 2011; Huart et al., 2013; Lie et al., 2021). Three major pathways within the NBA have been identified: the olfactory nerve pathway, the trigeminal nerve pathway, and the humoral pathway. These pathways provide structural bases for odor signals and exogenous substances to enter the CNS from the nasal cavity. To further clarify the functional characteristics of these pathways and their alterations under different physiological or pathological conditions, an increasing number of studies have adopted multimodal visualization technologies, enabling dynamic observations from the molecular to the tissue level. The following section provides a systematic overview of the anatomical connections of these pathways and visualization approaches used to study the NBA.

### Olfactory nerve pathway

The olfactory nerve represents the shortest and most direct neural connection between the CNS and the external environment. It is composed mainly of olfactory receptor neurons and exhibits three levels of projection. The olfactory epithelium consists of olfactory neurons, supporting cells, and basal cells. Olfactory neurons are bipolar neurons; one end extends a dendrite that divides into multiple cilia projecting into the mucus layer to interact with the external environment, while the other end extends an axon that crosses the basement membrane and is ensheathed by olfactory ensheathing cells. These axons ultimately converge to form the olfactory nerve, which then traverses the cribriform plate to reach the ipsilateral olfactory bulb, forming the primary projection connecting the olfactory system with the CNS (Crespo et al., 2019). Signals from the olfactory bulb are transmitted to the anterior olfactory nucleus, olfactory tubercle, piriform cortex, amygdala, and entorhinal cortex, forming secondary projections, and subsequently to widespread cortical and subcortical regions—including the orbitofrontal cortex, hippocampus, insula, anterior cingulate cortex, hypothalamus, brainstem, and even cerebellum—as tertiary projections (Soudry et al., 2011). These regions are highly interconnected and bidirectional, which is why familiar odors can evoke strong emotional responses or memory recall. This also explains why nasal pathologies can contribute to neurodegenerative diseases, anxiety, depression, and other CNS disorders (Fatuzzo et al., 2023).

Given the critical role of the olfactory nerve in the NBA, visualizing this pathway is essential for elucidating mechanisms and related applications. Traditional methods for monitoring the olfactory nerve include histology, electrophysiology, and tracer-based techniques. Ma et al. (2003) injected the lipophilic dye DiI into the mouse nasal septum and, after incubation, observed DiI transport along the olfactory nerve to the olfactory bulb, confirming the transmission pathway from the nasal cavity to the bulb. Another study has shown that olfactory nerve bundles and the olfactory bulb exhibit significant electrical changes following odor or chemical stimulation, highlighting the functional role of the olfactory nerve in signal transmission (Cang and Isaacson, 2003). However, these methods mostly rely on static or *ex vivo* tissue and lack dynamic whole-brain detection capability. In recent years, multimodal monitoring of the olfactory nerve has become a major research trend. Kurihara et al. (2022) used magnetic resonance imaging (MRI) combined with diffusion tensor imaging and diffusion tensor tractography to reconstruct a 3D projection map of the human olfactory nerve from the nasal olfactory epithelium to the olfactory bulb, revealing spatially organized mapping patterns within the bulb. Fa et al. (2010) applied manganese-enhanced magnetic resonance imaging and demonstrated that continuous olfactory stimulation after intranasal Mn^2+^ administration promoted manganese transport along the olfactory nerve to the olfactory bulb. Moreover, advances in whole-brain optical microscopy have enabled high-resolution visualization of neural structures at the microscopic level, and fluorescent labeling techniques allow 3D mapping of the olfactory nerve pathway (Liang and Luo, 2021). With progress in intranasal delivery technologies, fluorescent tracers, nanoparticles, and radiolabeled agents have been successfully used for *in vivo* visualization of the olfactory pathway, providing powerful tools for studying the structural and functional connectivity of NBA (Almahmoud et al., 2024). These methods enable dynamic, full-pathway monitoring of the olfactory system. Future studies may integrate chemogenetics or optogenetics to selectively inhibit or activate olfactory neurons and examine downstream changes in related brain regions, enabling comprehensive dynamic mapping of the olfactory nerve pathway.

### Trigeminal nerve pathway

The trigeminal nerve (cranial nerve V) is the largest cranial nerve in the head and neck and contributes to both sensory and motor functions. Its sensory fibers originate from the lateral root of the pons, with the main trunk forming the trigeminal ganglion within the cranial cavity. Three major branches arise from the ganglion: the ophthalmic nerve (V1), the maxillary nerve (V2), and the mandibular nerve (V3). The terminals of V1 and V2 extensively innervate the nasal mucosa and inferior turbinate. Therefore, the trigeminal nerve provides sensory input—including pain, temperature, touch, and burning sensations—from structures such as the nasal mucosa, sinuses, turbinates, and nasal septum (Yao and Sessle, 2018). The pathway primarily follows the route: peripheral nasal mucosa → branches of the trigeminal nerve → trigeminal ganglion → brainstem nuclei → thalamus/other relays → cerebral cortex. This pathway is one of the key routes for transmitting sensory information from the nose to the brain. Moreover, because trigeminal nerve terminals and olfactory nerve terminals in the nasal mucosa often occupy adjacent or interacting positions, cross-talk can occur. Under chemical odor stimulation, trigeminal-mediated perception and olfactory-mediated perception can complement each other.

The anatomical pathway of the trigeminal nerve not only enables extensive sensory transmission and partial reflexive motor regulation of the nasal mucosa but also constitutes a critical “high-speed route” directly connecting the peripheral nasal cavity to the CNS. This pathway can rapidly deliver inflammatory signals, pathogenic microorganisms, chemical irritants, and macromolecular drugs to the brainstem and higher-order brain regions within minutes or even shorter time frames (Johnson et al., 2010; Li et al., 2019). Therefore, dynamic monitoring of trigeminal nerve activity is highly important for elucidating its role and mechanisms within the NBA. Yang et al. (2022) applied intranasal HA/DP7-C for glioma treatment and, using *ex vivo* bioimaging, reported that HA/DP7-C/siRNA nanomicelles could be rapidly transported to the CNS via the trigeminal pathway within a few hours. Similarly, Johnson et al. (2010) demonstrated that after intranasal delivery of an infrared dye, signals can be detected in brain tissue within 10 minutes, with a distribution pattern highly consistent with the anatomical course of the trigeminal nerve in the nasal cavity. These studies rely primarily on fluorescent dyes or chemical tracers combined with histological sections, immunohistochemistry, fluorescence microscopy, and electron microscopy to visualize and track the trigeminal pathway. In recent years, radioactive tracers or MRI contrast agents (e.g., gadolinium [Gd]) for *in vivo* dynamic imaging have been used to observe the temporal and spatial distributions of tracers from the nasal cavity to the cranial cavity (Almahmoud et al., 2024). Another study introduced the DCLNet model, which achieves high-precision automatic segmentation of the trigeminal nerve by integrating multimodal information from structural MRI and diffusion MRI and by employing a co-learning strategy that combines coarse labels with fine manual annotations. This approach effectively incorporates both neural fiber trajectory information and anatomical structural features (Xie et al., 2025a). Moreover, a recent fiber-tracking–based study constructed a multi-parameter, multi-stage diffusion MRI atlas that enables the automatic mapping of several major cranial nerves. By analyzing the nerve fiber bundles, the authors identified bundles with consistent anatomical structures. These bundles demonstrated a high degree of spatial correspondence with expert annotations across multiple datasets. This atlas provides a standardized and reproducible *in vivo* framework for cranial nerve reconstruction, offering a valuable tool for the quantitative assessment of neural pathways involved in nose–brain communication—particularly the trigeminal nerve branches associated with nasal sensory and transport pathways (Xie et al., 2025b). The latest strategies also include intranasal injection of nanoparticles and tracking their crossing of the NBA, migration routes, and final accumulation sites, enabling dynamic detection of the trigeminal pathway (Zha et al., 2022). Veronesi et al. (2021) systematically investigated the whole-body and brain distribution patterns of intranasally administered nanoparticles by delivering radiolabeled nanoparticles into the nasal cavity of rats and combining positron emission tomography/CT imaging. This work provides quantitative methods and experimental evidence for intranasal drug delivery, particularly for brain-targeted delivery (Veronesi et al., 2021). Future studies urgently need to further develop multimodal monitoring technologies and optimize tracer molecules and imaging methods to achieve higher spatiotemporal resolution in monitoring the dynamic processes of the trigeminal nerve within the NBA.

### Humoral pathway

In recent years, the discovery of the brain lymphatic system has provided a new theoretical framework for understanding CSF circulation and CNS waste clearance. After being produced by the choroid plexus, CSF circulates within the ventricles and subarachnoid space, eventually flowing into the dural venous sinuses and mixing with venous blood (Proulx, 2021). As CSF passes through the cribriform plate region, it can enter the lamina propria of the olfactory mucosa via perivascular spaces near olfactory nerve fibers and subsequently drain into the nasal lymphatic system, enabling material exchange between peripheral and central fluids. This pathway not only facilitates the clearance of metabolic waste from the brain but also provides a potential route for intranasal drug delivery targeting CNS disorders (Norwood et al., 2019). However, current research on this pathway has been conducted mostly in animals, and there are significant differences across species.

Substantial experimental evidence further supports the existence of this pathway. In one study, high-contrast Evans blue (EB) was injected into the cisterna magna of mice, successfully visualizing CSF drainage pathways. EB exists in CSF as a free, low-molecular-weight tracer, and its penetration into the nasal lymphatic system demonstrated that CSF can enter the nasal lymphatic pathway via the cribriform plate (Norwood et al., 2019). In another multi-species cadaver tracing experiment, Johnston et al. (2004) injected a yellow tracer into the subarachnoid space. The tracer was detected in the nasal cavity, as well as in the nasal mucosa and turbinate tissues, across monkeys, mice, rats, pigs, and humans, indicating the conserved nature of this pathway among species. Zhu et al. (2025) employed a 3D MR-fingerprinting dynamic contrast-enhanced scanning method to achieve, for the first time in mice, whole-brain dynamic tracking of the intracerebral distribution of a contrast agent (Gd-DTPA). These findings revealed the transport kinetics of CSF/interstitial fluid across different brain regions. This experimental approach holds potential for future applications in dynamically tracking and even quantitatively assessing fluid pathways along the NBA. Although these studies highlight the critical role of the cribriform plate in CSF outflow to the nasal lymphatic system, the actual physiological flow rate remains unclear. Moreover, owing to limitations in human samples and insufficient resolution of *in vivo* imaging techniques, dynamic visualization studies of this pathway are relatively scarce, leaving considerable uncertainty regarding the extent to which nasal substances or drugs can effectively reach the CNS via this route. Future research should focus on improving the visualization and quantification of the nasal–brain lymphatic pathway, particularly through high-resolution imaging technologies such as high-field MRI, dynamic contrast-enhanced MRI, and positron emission tomography tracers, to monitor the cribriform plate region and meningeal lymphatic system in real time. A systematic evaluation of whether different molecules and nanocarriers can traverse this pathway to enter the CNS, along with optimization of intranasal delivery strategies to increase trans-cribriform efficiency, is needed. Additionally, exploring the diagnostic and clinical potential of this pathway in neurological disorders represents an important future direction. The visualization techniques for the three major pathways of the nose–brain axis are summarized in **Table 1**.

**Table 1:** Visualization techniques for the nose–brain axis

Pathway	Method	Mechanism	Reference
Olfactory nerve	DiI tracing	DiI diffuses anterogradely along olfactory nerve fibers, labeling corresponding layers of the olfactory bulb	Ma et al., 2003
	Electrophysiology	Odor or chemical stimulation induces significant electrophysiological changes in olfactory nerve bundles and the olfactory bulb	Cang and Isaacson, 2003
	MRI & DTI & DTT	3D reconstruction of olfactory nerve fibers from the nasal olfactory epithelium to the olfactory bulb	Kurihara et al., 2022
	Manganese-enhanced magnetic resonance imaging	MRI observations showed that continuous olfactory stimulation after intranasal administration of Mn2+ can enhance the transport of manganese along the olfactory nerve to the olfactory bulb	Fa et al., 2010
	Whole-brain optical microscopy & fluorescent labeling	Direct three-dimensional mapping of the olfactory nerve pathway	Liang and Luo, 2021
Trigeminal nerve	HA/DP7-C/siRNA nanomicelles	Ex vivo bioimaging shows transport occurs via the trigeminal nerve	Yang et al., 2022
	Infrared dye	Dye distribution closely matches the anatomical course of the trigeminal nerve in the nasal cavity	Johnson et al., 2010
	Radioactive tracers or MRI contrast agents	In vivo radiologic imaging	Almahmoud et al., 2024
	Structural MRI & diffusion MRI	Analysis and integration of neural fiber tract information with anatomical structural features	Xie et al., 2025
	Radiolabeled nanoparticles & PET/CT	PET/CT imaging to assess distribution of radiolabeled nanoparticles	Veronesi et al., 2021
Humoral	EB	EB serves as a tracer in cerebrospinal fluid and can penetrate into the nasal lymphatic system	Norwood et al., 2019
	Yellow tracer	After injection into the subarachnoid space, the tracer is detected in the nasal cavity, nasal mucosa, and turbinate tissues	Johnston et al., 2004

CT: Computed tomography; DTI: diffusion tensor imaging; DTT: diffusion tensor tractography; EB: evans blue; MRI: magnetic resonance imaging; PET: positron emission tomography.

Thus, the NBA primarily mediates communication between the nasal cavity and the brain through neural and fluidic pathways, which differ markedly in their mechanisms, timing, and molecular dependence. The neural pathway relies on substances being recognized and endocytosed by the terminals of the olfactory or trigeminal nerves, followed by rapid axonal transport directly into the brain. This process completely bypasses the BBB and is relatively fast, allowing small molecules to be transported more efficiently, whereas the transport efficiency of larger molecules is reduced. Moreover, delivery via this pathway is often precisely mapped to brain regions corresponding to the innervating nerves, providing potential for targeted delivery in the future. In contrast, the fluidic pathway depends on substances being absorbed through the nasal mucosa into the bloodstream or CSF circulation. This process is slower, favors lipophilic molecules, and results in a whole-brain distribution with relatively weak targeting. Overall, the fluidic pathway formed by the brain lymphatic system constitutes the “slow route” of the NBA, complementing the “fast route” mediated by the olfactory and trigeminal nerves, together forming a comprehensive nose–brain communication network (Lochhead and Thorne, 2012).

## Mechanisms by Which Nasal Inflammation Affects the Central Nervous System via the Nose–Brain Axis

Traditional studies on nasal inflammation have focused primarily on local nasal symptoms and immunological mechanisms. However, recent evidence suggests that nasal inflammation is not confined to the respiratory tract and may significantly impact CNS function. Studies indicate that bacterial infection or allergen exposure in the nasal mucosa can lead to the release of cytokines, histamine, neuropeptides, and neurotransmitters, triggering local inflammation and transmitting signals to the CNS via neural pathways. This process may contribute to CNS disorders such as depression, anxiety, migraine, and neurodegenerative diseases (Amritwar et al., 2017; Ramsridhar, 2023). Additional studies have shown that during neuroinflammation, immune cells surrounding olfactory nerve fibers increase significantly, and these cells can target microbes or bacteria in both the nasal cavity and the CNS (Laaker et al., 2025). This has led to the concept of neuroimmune interactions, wherein the communication between the nasal cavity and CNS involves not only neural signaling but also immune system participation, including mucosal immunity, microbial immune responses, mucosal barriers, and cytokine release. Nasal inflammation activates inflammatory cells on the nasal mucosal surface, resulting in the release of histamine, leukotrienes, prostaglandins, and multiple cytokines (e.g., interleukin [IL]-4, IL-6, tumor necrosis factor-α [TNF-α], and IL-1β). These inflammatory mediators can enter the nervous system via the olfactory nerve, trigeminal nerve, or systemic circulation, activating microglia and astrocytes and inducing the intracranial production of cytokines (e.g., IL-1β, IL-18, IL-6, and TNF-α), leading to neurotoxicity, neuronal damage, and pathological changes in CNS regions, ultimately contributing to CNS disorders (Lakatos and Rosta, 2025). Experimental evidence supports this mechanism. A recent study explored the mechanism by which NLRP3 exerts its function. This study demonstrated that upon activation, the central NACHT domain of NLRP3 undergoes a conformational change, triggering the release of the pro-inflammatory cytokines IL-1β and IL-18 and inducing pyroptosis. Moreover, treatment with an NLRP3 inhibitor leads to conformational remodeling in key domains of the protein, placing NLRP3 in a conformation that is less prone to activation (Casali et al., 2023). Similarly, another study used molecular simulations to construct an NLRP3–NEK7–ADP system, reproducing most structural features of the NLRP3 inflammasome. Their results indicated that removal of NEK7 caused the inflammasome to shrink and adopt a more compact conformation, suggesting a stable state, whereas substitution of the cofactor from ADP to ATP resulted in a more open conformation, implying a transition to a readily activatable state (El-Sayed et al., 2022). Collectively, these studies demonstrate that the function of NLRP3 is dependent on conformational changes. Similarly, Ebrahim Soltani et al. (2022) reported that AR activates the TLR4–NF-κB signaling pathway, increasing the levels of TNF-α, IL-1β, and IL-6 in the olfactory bulb and impairing olfactory function. TAK-242, a TLR4 inhibitor, suppressed olfactory bulb neuroinflammation and significantly alleviated AR-induced olfactory dysfunction. AR mice exhibit elevated expression of TLR4, NF-κB, IL-1β, and TNF-α in the hippocampus and plasma proinflammatory cytokines, along with demyelination, increased cell death, and M1 macrophage aggregation in the hippocampus. These findings collectively confirm that chronic nasal inflammation can induce CNS neuroinflammation via the olfactory and trigeminal pathways, although the detailed mechanisms require further investigation. In addition to the classical pathways mentioned above, other neuroimmune mechanisms also warrant attention. For example, the α7 nicotinic acetylcholine receptor, which is expressed primarily in microglia, astrocytes, and neurons, plays a central role in the cholinergic anti-inflammatory pathway. Nasal stimulation can activate both astrocytes and microglia, suggesting that the α7 nicotinic acetylcholine receptor may also be involved in NBA. Moreover, the sirtuin/AMPK pathway, a key regulator of cellular energy metabolism and stress responses, may inhibit the NLRP3 inflammasome upon activation and alleviate neuroinflammation. These targets offer new directions for interventions in neuroimmune dysfunction mediated by the NBA. Nonetheless, the involvement of these two pathways in NBA has not been empirically established. Future investigations could focus on these routes, potentially offering new insights into neuroimmune dysregulation mediated by NBA.

In addition, peripheral nasal inflammation, such as allergic rhinitis (AR), often causes goblet cell hyperplasia and excessive secretion. However, allergen or inflammatory mediator exposure does not directly trigger goblet cell degranulation. A new mechanism has been proposed: stimulation of nerve terminals by allergens or inflammatory mediators induces the expression of neuropeptides such as substance P (SP), calcitonin gene-related peptide (CGRP), nerve growth factor, and neuromedin U (Xu et al., 2020). Mosimann et al. (1993) reported elevated levels of SP, CGRP, and VIP in nasal lavage fluid in chronic nasal inflammation models, supporting the expression of neuropeptides during chronic nasal inflammation. These neuropeptides mediate neuroimmune responses by activating specific receptors on immune cells, leading to neural remodeling and functional modulation. Collectively, these studies demonstrate that neuroimmune interactions contribute to the effects of nasal inflammation on the nervous system. Dysregulation of this neuroimmune network can further disrupt the levels or functions of neurotransmitters such as histamine, serotonin (5-HT), norepinephrine, γ-aminobutyric acid, and dopamine (Liu et al., 2021; Klimov et al., 2022). These molecules can directly affect peripheral tissues and activate sensory nerve terminals, transmitting signals to the CNS. Chronic dysregulation may also impact the circulatory system, exacerbating neurological symptoms. For example, Yang et al. (2022a) reported that serum 5-HT levels are higher in AR patients than in healthy controls, with a positive correlation between serum 5-HT and AR-related cytokines. Overall, these studies indicate that the effects of nasal inflammation on the CNS result from neuroimmune interactions mediated by NBA. This neuroimmune network plays a coordinated, rapid-response, and protective regulatory role in CNS function, and its dysregulation may be a key mechanism underlying the development of multiple CNS disorders.

A recent study has shown that allergic airway inflammation significantly affects hippocampal synaptic plasticity (Klein et al., 2016). In addition, Hasegawa-Ishii et al. (2017) induced persistent nasal inflammation in mice through repeated intranasal administration of LPS, which after three weeks resulted in the loss of olfactory sensory neurons, glial proliferation, and synaptic loss in the olfactory bulb. Moreover, even following therapeutic intervention, neuronal regeneration remained incomplete. These findings suggest that nasal inflammation can alter neural plasticity and impair neuroregeneration via the NBA, which may represent a key mechanism linking nasal inflammation to neurodegenerative diseases.

Nasal inflammation activates astrocytes and microglia, leading to elevated expression of inflammatory cytokines. These cytokines can cross the BBB or act on the brain through nose–brain immune pathways, thereby disrupting neurogenesis. Furthermore, inflammatory cytokines released during nasal inflammation can enter the CNS via the NBA and subsequently cause nasal lymphatic–CSF drainage dysfunction, microbiome dysbiosis, and abnormal neural stem cell differentiation, all of which are closely associated with neurogenesis (Saikarthik et al., 2022; Xie et al., 2022; Chae et al., 2024b; Vásquez-Pérez et al., 2025). The work of Guzzetta et al. (2022) provides a comprehensive and systematic summary of the crucial role of the gut microbiota in regulating neurogenesis. The microbiota shapes neurogenesis primarily by modulating central microglial activity and peripheral immune states, thereby influencing neural stem cell proliferation and survival. Silva et al. (2020) demonstrated that microbial metabolites—such as short-chain fatty acids—can cross the BBB to promote neuronal differentiation. In addition, the gut microbiota communicates via neuroimmune pathways and regulates endocrine function, neurotrophic factors (e.g., brain-derived neurotrophic factor), and the hypothalamic–adrenal axis, collectively shaping hippocampal neurogenesis. Impaired nasal lymphatic–CSF drainage can hinder the clearance of metabolic waste from the brain, leading to neuropathological changes. Because neurogenesis is closely linked to AD, understanding how nasal lymphatic–CSF drainage interacts with neurogenic processes is critical (Phillips and Schwartz, 2024). Ribeiro et al. (2021) demonstrated that activation of the adenosine A₂A receptor promotes the self-renewal of hippocampal stem cells and reduces the death of newborn neurons and their progenitors. Taken together, nasal inflammation may influence neurogenesis through multiple interconnected mechanisms, thereby indirectly contributing to neurological disorders. These insights also highlight the potential of NBA-mediated intranasal drug delivery as a mechanistic and etiological therapeutic strategy for CNS diseases.

## Nose–Brain Axis: A Bridge for Cross-System Pathogenesis and Therapy

The existence of NBA allows direct communication between the nasal cavity and the brain. Through neuroimmune interactions, chronic nasal inflammation can affect the CNS. Peripheral nasal inflammation, such as AR, can not only activate microglia and astrocytes via the NBA, triggering neuroinflammation and directly impacting the CNS but also influence CNS neurogenesis indirectly through alterations in the microbial environment, impairment of nasal lymphatic–CSF drainage, and changes in neural stem cell activity. These processes may contribute to CNS disorders, including mood disorders, migraines, PD, and AD. Meanwhile, intranasal drug delivery has emerged as a novel therapeutic approach that can deliver drugs via the NBA, bypass the BBB, improve intracranial bioavailability, and shorten onset time, thus providing new opportunities for crosssystem treatment. However, since the repair of neural structure and function is the ultimate goal of CNS disease therapy, NBA-mediated strategies should not be limited to symptom relief but should also promote neural regeneration to achieve etiological treatment. Accordingly, recent research has increasingly focused on neural regeneration as a means to fundamentally treat various CNS disorders, employing stem cells, exosomes, neurotrophic factors, and other pharmacological agents. To achieve efficient delivery of these therapeutics, many studies have utilized nanoparticles or liposomes as carriers.

### Emotional disorders

Anxiety and depression are common mental health problems among children, adolescents, and adults and represent major contributors to the global health burden (Lu et al., 2024). Increasing epidemiological and clinical evidence indicates a close association between AR and a greater risk of depression and anxiety. One study reported that individuals with AR have an approximately 1.9-fold greater likelihood of developing anxiety and a 1.6-fold greater likelihood of developing depression compared to the general population (Rodrigues et al., 2021).

Basic research has shown that AR can lead to decreased central 5-HT levels, reduced γ-aminobutyric acid activity, and abnormal glutamate/norepinephrine levels, which impair emotion regulation and inhibitory circuits, resulting in anxiety-like behaviors (Chen et al., 2006; Arora et al., 2024; Yuan et al., 2025). In addition to these neurotransmitter changes, neuroimmune interactions play an even more critical role in anxiety development. AR induces the activation of numerous eosinophils, mast cells, and Th2 cells in the nasal mucosa, which secrete cytokines such as IL-4, IL-13, IL-1β, and IL-6. Some of these cytokines can travel along the NBA to activate microglia and astrocytes, exacerbating intracranial neuroinflammation and leading to neural remodeling in the cingulate cortex and amygdala—key regions driving anxiety and depression (Gholami-Mahtaj et al., 2022). Gao et al. (2023) demonstrated that TET2 may increase IL-18 and IL-1β in the ACC by activating NLRP3, inducing neuroimmune remodeling in the ACC and contributing to anxiety- and depression-related symptoms in patients with AR. Mou et al. (2024a) reported that microglial P2X7 receptors in the olfactory bulb play a pathogenic role in AR-related emotional disorders; blocking P2X7 or inhibiting microglial activation alleviates behavioral abnormalities and local neuroinflammation.

Intranasal drug delivery has made significant progress in the treatment of depression- and anxiety-like behaviors. Previous studies have confirmed that intranasal administration of oxytocin significantly reduces depressive behaviors and promotes hippocampal neurogenesis (Ji et al., 2016). In a rat model of anxiety, animals receiving intranasal neuropeptides exhibited lower anxiety-like behaviors and reduced depressive-like behaviors compared to controls (Serova et al., 2014). Recently, a study reviewing 33 patients with major depressive disorder treated with intranasal esketamine reported that approximately 70% of patients responded to the treatment, with overall good tolerability (Abalde García et al., 2025). In animal experiments, intranasal administration of mesenchymal stem cell-derived exosomes was shown to regulate autophagy and inflammatory pathways (e.g., AMPK/mTOR), significantly improving depressive-like behaviors and reducing anxiety tendencies in mice (Sun et al., 2025). Another study utilized AAV9-mediated CRISPR-Cas9 via intranasal delivery to reduce 5-HT2A receptor expression in the brain, significantly alleviating anxiety-like behaviors (Rohn et al., 2023). Additionally, intranasal delivery of brain-derived neurotrophic factor protein in mice reduces proinflammatory cytokine expression in the amygdala, decreases oxidative stress, and increases hippocampal neurogenesis, thereby improving depressive behaviors (Li et al., 2022). These findings provide new avenues for cross-system modulation of emotional disorders. Furthermore, intranasal drug delivery allows drugs to be encapsulated in nanoparticles or liposomes, increasing CNS bioavailability, accelerating onset, and reducing systemic side effects (Nguyen and Duong, 2025b). Tatiana et al. designed a cationic hybrid liposome to increase the efficiency of drug delivery to the brain via the nasal mucosa and demonstrated that compared with intravenous injection, intranasal administration significantly increased the brain bioavailability of the carrier (Pashirova et al., 2018).

Despite the promising potential of intranasal drug delivery for treating anxiety and depression, this approach has inherent limitations. Issues such as nasal retention time, drug distribution, and the need for repeated administration in chronic conditions remain unresolved. In particular, for innovative therapies involving gene editing or biologics, immunogenicity and long-term safety continue to pose major obstacles to clinical translation.

### Migraine

Migraine is a complex neurological disorder characterized by recurrent headaches, often accompanied by sensory disturbances, nausea, and autonomic symptoms (Burstein et al., 2015). Although its precise pathophysiological mechanisms are not fully understood, current research suggests that migraine results from a combined vascular–neural mechanism (Frimpong-Manson et al., 2024). AR can lead to increased histamine release, which has vasodilatory effects and is considered a key pathological factor in migraine pathogenesis. Furthermore, owing to the presence of the trigeminal nerve pathway within the NBA—and the well-established role of trigeminal activation in migraine—AR may trigger migraines by activating the trigeminal nerve via the NBA. Increasing amounts of epidemiological evidence support a significant association between migraine and allergic diseases. Pai and Rosario (2018) reported that among 150 AR patients, approximately 68% experienced migraines, with greater headache severity observed in females and individuals with elevated IgE levels. Similarly, Ku et al. (2006) reported that 34% of 76 AR patients had migraines, whereas only 4% of 57 non-AR individuals did, indicating that the likelihood of migraine in the AR group was 14.3 times greater than that in the non-AR group. Although numerous studies have demonstrated a strong association between AR and migraine, research on the causal relationship between the two remains limited. Future studies should not only clarify the relationship but also investigate the underlying mechanisms and potential causality between AR and migraine.

Mainstream migraine treatments include acute pain relief and preventive strategies to reduce attack frequency. Commonly used medications include NSAIDs, acetaminophen, and triptans, while emerging drugs include CGRP receptor antagonists (Peck et al., 2020; Anukoolwittaya et al., 2025). Intranasal drug delivery, with its rapid onset, high bioavailability, and ability to bypass the gastrointestinal tract, is emerging as a novel approach for migraine treatment. A phase III clinical trial demonstrated that intranasal sumatriptan powder significantly increased pain relief within 0.5–2 hours compared with placebo, and the nasal formulation achieved faster onset than did oral administration (Al-Salama and Scott, 2016). However, triptans cause vasoconstriction and are not suitable for patients with a history of cardiovascular or cerebrovascular diseases. Saba et al. developed a nasal formulation of lasmiditan and applied it in rabbits, which demonstrated faster absorption and higher bioavailability than did oral administration (Jabir and Rajab, 2025). Another study developed a novel mucoadhesive cubic in situ nasal gel combined with sumatriptan succinate for migraine treatment, which resulted in significantly higher brain concentrations of sumatriptan than intravenous injection (Tekade et al., 2023). Recent research on intranasal CGRP receptor antagonists revealed that 2 hours after administration, migraine symptom relief was markedly superior to that of placebo (Lipton et al., 2023). Additionally, clinical trials indicate that intranasal dihydroergotamine powder provides bioavailability and onset comparable to or better than traditional sprays, with greater convenience than subcutaneous or intravenous administration (Albrecht et al., 2020). Experimental approaches targeting nasal neural pathways, such as intranasal lidocaine anesthesia, have also been applied for migraine treatment (Chi et al., 2019). These studies collectively suggest that intranasal drug delivery, leveraging the rich nasal blood supply and direct connections to the trigeminal and olfactory nerve pathways, can rapidly enter systemic circulation or reach CNS targets via the NBA while bypassing gastrointestinal absorption and avoiding issues such as vomiting or delayed gastric emptying. Research has increasingly focused on the use of nasal nanoparticles and liposomes as carriers to increase drug stability and absorption. However, most studies remain at the animal or early-stage level, and toxicity assessments of nanocarrier systems are still insufficient, presenting challenges for clinical translation.

**Figure 1:**
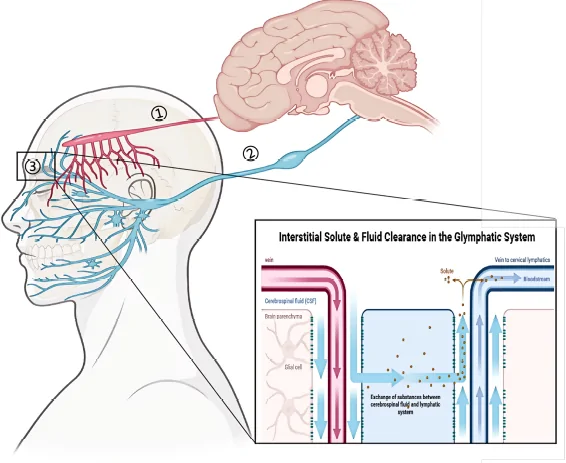
Anatomical pathways through which the nose–brain axis exerts its effects: (1) Olfactory nerve pathway; (2) trigeminal nerve pathway; (3) humoral pathway. Created with BioRender.com.

### Parkinson’s disease

PD is a chronic neurological disorder characterized by a wide range of motor and nonmotor symptoms and complex pathophysiological mechanisms. Neuroinflammation leading to α-synuclein (α-syn) aggregation is considered a key factor in PD progression (Fan et al., 2020). The olfactory bulb not only serves as a critical regulatory node for immune and inflammatory entry into the brain but also acts as an initiation site for α-syn aggregation and propagation (Rey et al., 2016). Therefore, nasal inflammation may be closely related to PD onset. Nam et al. (2024) surveyed 398,936 participants aged 40 years and older and reported that individuals with AR had a greater risk of developing PD than those without AR. Currently, most studies on the relationship between nasal inflammation and PD are clinical, whereas animal experiments and mechanistic studies are limited. Therefore, we speculate that AR may activate microglia and astrocytes, leading to the release of inflammatory mediators such as TNF-α and IL-1β. These substances can exacerbate neuronal damage and death, thereby accelerating the progression of neurological disorders such as PD. Future research should focus on investigating the causal relationship between nasal inflammation and PD, as well as elucidating the underlying mechanisms.

Current PD treatments primarily include dopamine replacement therapy and surgical interventions. While dopamine replacement can improve motor symptoms and enhance patient quality of life, it does not prevent neurodegenerative changes. Surgical treatments, which involve the implantation of electrodes in relevant brain regions to modulate abnormal neural circuit activity, can reduce medication use but are costly and may induce side effects such as depression. With the aging population, the number of PD patients is increasing, yet disease-modifying therapies remain unavailable, highlighting the urgent need to develop treatments capable of altering disease progression. Currently, first-line PD medications are administered orally, but the BBB limits their CNS bioavailability. Intranasal delivery, however, allows drugs to reach the brain directly via the olfactory and trigeminal nerve pathways, bypassing the BBB and substantially improving bioavailability. Kim et al. (2009) compared oral and intranasal formulations in rats and reported that intranasal sprays achieved faster and greater L-DOPA exposure in brain tissue, supporting the superior bioavailability of intranasal administration. Sridhar et al. (2018) demonstrated that, compared with oral administration, intranasal administration resulted in 20-fold higher concentrations of selagin in the brain and 12-fold higher concentrations in plasma. These studies highlight the clear advantages of nasal drug delivery over oral administration in the treatment of PD. Next, we summarize the latest research progress on intranasal administration for PD treatment.

The primary pathological hallmark of PD is the aggregation and propagation of α-syn. Additionally, abnormal interactions between α-syn and oxidized lipid metabolites lead to mitochondrial dysfunction. Therefore, therapeutic strategies for PD mainly focus on reducing α-syn aggregation and improving lipid metabolism abnormalities. Studies have shown that small interfering RNAs (siRNAs) can knock down α-syn, prompting the development of nanoparticle-mediated intranasal siRNA delivery systems, which significantly reduce α-syn levels in the brain and offer a novel therapeutic approach for PD (Feja et al., 2025). To address the mitochondrial dysfunction caused by lipid metabolic abnormalities, insulin—known to improve mitochondrial function—has been delivered intranasally. In PD rat models, intranasal insulin administration markedly improved motor deficits, with a uniform distribution observed along the trigeminal nerve (Lochhead et al., 2019; Iravanpour et al., 2021). Furthermore, to mitigate motor deficits caused by dopaminergic neuron loss, studies have employed olfactory ectomesenchymal stem cells delivered intranasally. In PD rat models, this approach increased the levels of dopaminergic neuron markers in relevant brain regions and substantially improved motor symptoms (Simorgh et al., 2021). A highly innovative study in PD rat models revealed that mitochondria are delivered to the brain via the intranasal route, resulting in reduced inflammatory cytokines, increased dopaminergic neuron survival, and improved motor function (Chang et al., 2021). Additionally, a recent study combined dental pulp stem cell-derived extracellular vesicles with the natural antioxidant phloroglucinol and administered them intranasally, which not only improved motor deficits but also promoted neuroregeneration, suggesting a potential disease-modifying strategy for PD (Mondal et al., 2025). Future research should systematically evaluate the safety and efficacy of intranasal delivery strategies in PD patients, explore the synergistic effects of combined interventions, and integrate imaging and biomarker monitoring to facilitate translation from symptomatic relief to disease-modifying therapies.

### Alzheimer’s disease

In recent years, increasing epidemiological evidence suggests a potential association between nasal inflammation and cognitive decline, as well as the risk of AD. Understanding this relationship holds important etiological significance and may inform clinical prevention strategies for AD. From an epidemiological perspective, several large prospective cohort studies have reported a positive correlation between allergic diseases and an increased future risk of dementia. Joh et al. (2023) followed 260,705 dementia cases over eight years and concluded that allergic diseases were positively associated with dementia risk. Compared with individuals without allergic diseases, patients with AR had a multivariable hazard ratio of 1.10 for dementia. Wang et al. (2024) conducted a genome-wide association study including 66,645 patients with allergic diseases and 12,281 dementia cases, demonstrating a potential causal link between AR and increased AD risk. From a biological mechanism perspective, current research on AR and AD mainly involves the following hypotheses: (1) NBA is a bridging pathway. During allergic responses, peripheral pro-inflammatory cytokines (e.g., IL-4, IL-6, IL-13, and TNF) are chronically elevated. Through neuroimmune interactions along the NBA, central immune cells are activated, inducing neuroinflammation and promoting amyloid-β and tau pathology or exacerbating neurodegeneration. (2) Inflammation-induced BBB permeability: Peripheral immune cytokines may enter the CNS, triggering neuroinflammation. Chronic peripheral inflammation can be transmitted to the CNS, altering the immune status of the brain, activating astrocytes, and promoting neurodegenerative pathways. In their study, allergic mice presented elevated brain IgG and IgE levels along with increased tau phosphorylation (Sarlus et al., 2012). However, this study did not clarify the mechanism by which AR induces tau phosphorylation.

Currently, therapeutic strategies for AD primarily focus on inhibiting tau aggregation and modulating neurotransmitter imbalances. In recent years, intranasal drug delivery has emerged as a novel brain-targeting approach, becoming a hotspot in AD research. Multiple intranasal (IN) delivery strategies, including antioxidants, metabolic pathway modulators, anti-neuroinflammatory drugs, immunotherapies, anti-tau antibodies, small-molecule drug repurposing, and peptide-based therapeutics, have been explored. In antioxidant studies, an Aβ-induced oxidative stress and neuroinflammation model demonstrated that continuous intranasal delivery of glutathione significantly reduced oxidative product formation and suppressed microglial activation, thereby preventing cognitive impairment. Notably, brain glutathione levels are approximately four times higher than systemic levels, indicating that IN delivery effectively elevates brain antioxidant concentrations and may delay AD onset (Bhilare et al., 2025). With respect to tau regulation, a previous study has shown that IN delivery of bambuterol reduces tau hyperphosphorylation in hippocampal neurons exposed to Aβ toxicity while preserving synaptic structure and function, preventing long-term spatial memory deficits (Groo et al., 2025). Furthermore, researchers developed a tau aggregate-specific antibody, TTCM2, encapsulated in microparticles for IN administration. Experiments have demonstrated that this approach allows more efficient brain entry, clears toxic tau, and improves cognitive performance (Gaikwad et al., 2024). In immune modulation, IN application of CD3 antibodies also showed potential therapeutic effects in AD. Nasal delivery of CD3 antibodies restored microglial M0 phenotypes and improved cognitive behaviors in AD animal models, suggesting its role in modulating neuroinflammation for AD therapy (Lopes et al., 2023). Additionally, experimental studies indicate that IN insulin can regulate multiple AD-related signaling pathways, including those that promote Aβ clearance, reduce tau phosphorylation, and enhance synaptic plasticity. IN insulin also increases brain glutathione levels, thereby improving cognitive deficits (de Oliveira Andrade et al., 2025; Kawashima et al., 2025). In summary, IN drug delivery enables efficient brain-targeted therapy and exerts multifaceted effects through antioxidant action, tau aggregation inhibition, neuroinflammation modulation, and metabolic pathway regulation, offering novel strategies and theoretical support for AD prevention and treatment. However, most IN approaches remain at the experimental stage, and clinical translation faces challenges. Future studies should focus on optimizing drug formulation, dosage, delivery frequency, and long-term safety, in combination with targeted strategies, to achieve precise prevention and therapy. The cross-system therapeutic strategies and corresponding mechanisms for common CNS diseases via the nose–brain axis are summarized in **Table 2**.

**Table 2:** Cross-system therapeutic strategies and mechanisms for various central nervous system diseases

Disease	Drug/Formulation	Mechanism	Reference
Emotional disorders	Oxytocin	Promote hippocampal neurogenesis	Ji et al., 2016
	mesenchymal stem cell-derived exosomes	Regulate autophagy and inflammatory pathways	Sun et al., 2025
	CRISPR -Cas9	Reduce 5-HT2A receptor expression in the brain	Rohn et al., 2023
	BDNF	Reduce proinflammatory cytokine expression in the amygdala, decrease oxidative stress, and increase hippocampal neurogenesis	Li et al., 2022
Migraine	Sumatriptan	Constriction of previously dilated intracranial/extracranial blood vessels.	Al-Salama and Scott, 2016
	Lasmiditan	Inhibit the release of neuropeptides from trigeminal nerve terminals	Jabir and Rajab, 2025
	Calcitonin gene-related peptide receptor antagonists	Inhibit the release of CGRP	Lipton et al., 2023
	Dihydroergotamine	Constriction of blood vessels and inhibition of neuroinflammatory signaling	Albrecht et al., 2020
	Lidocaine	Local anesthetic effect and neural signal suppression	Chi et al., 2019
PD	L-Dopa	Cross the blood–brain barrier and generate dopamine in the brain	Kim et al., 2009
	Selegiline	Reduce the degradation of dopamine in the brain	Sridhar et al., 2018
	siRNAs	Knockdown α-synuclein	Feja et al., 2025
	Insulin	Improve mitochondrial function	Lochhead et al., 2019; Iravanpour et al., 2021
	Olfactory ectomesenchymal stem cells	Ameliorate dopaminergic neuron loss	Simorgh et al., 2021
	Mitochondria	Reduce inflammatory cytokines; increase dopaminergic neuron survival	Chang et al., 2021
	Dental pulp stem cell-derived extracellular vesicles & natural antioxidant phloroglucinol	Promote neuroregeneration	Mondal et al., 2025
AD	Glutathione	Reduce oxidative product formation and suppress microglial activation	Bhilare et al., 2025
	Bambuterol	Reduce tau hyperphosphorylation in hippocampal neurons exposed to Aβ toxicity preserving synaptic structure and function	Groo et al., 2025
	TTCM2	Reduce tau-aggregate	Gaikwad et al., 2024
	CD3 antibodies	Restored microglial M0 phenotypes	Lopes et al., 2023

5-HT: Serotonin; AD: Alzheimer’s disease; BDNF: brain-derived neurotrophic factor; CGRP: calcitonin gene-related peptide; PD: Parkinson’s disease

## Challenges in Clinical Translation

In recent years, research on intranasal drug delivery for treating CNS diseases via the NBA has made significant progress. This approach has advantages such as bypassing the BBB, precise delivery, rapid onset, high bioavailability, and minimal systemic side effects. However, most published studies remain limited to animal experiments, with no unified standards for administration frequency, carriers, or dosage. Moreover, the olfactory region and nasal anatomy in animals differ significantly from those in humans, making translation of dosing, delivery efficiency, and drug metabolism to humans challenging. Systematic studies quantifying brain drug concentrations, target region distributions, and adverse reactions are rare, and research on neurotoxicity from long-term, repeated administrations for chronic diseases is even more limited. Repeated nasal administration may also cause nasal mucosal irritation, changes to the fibrous mucosal barrier, and immunogenic alterations, which in turn affect drug delivery efficiency. Individual anatomical differences in the nasal cavity are a major factor affecting the reproducibility of nasal drug delivery. Significant variations exist among individuals in nasal passage width, turbinate size, nasal curvature, and branching angles of the upper respiratory tract. These structural differences directly influence fluid dynamics and the local deposition patterns of drugs in the nasal cavity. Additionally, mucociliary clearance ability varies between individuals and is affected by age, sex, environmental factors, and disease status (Cellina et al., 2020; Chen et al., 2024). The activity of the mucociliary system not only affects the residence time of drugs on the nasal surface but also affects the probability of drug transport to the lower respiratory tract, thereby altering local and systemic exposure. Furthermore, variations in the location and area of the olfactory region can significantly impact the efficiency of drug transport to the CNS via the olfactory nerve pathway. Individuals with larger and more exposed olfactory regions have greater nose-to-brain drug penetration efficiency (Vishnumurthy et al., 2025). Factors such as mucosal thickness, humidity, and blood flow in the nasal cavity also influence drug absorption and transport. In addition, the spray angle, droplet size, spray velocity, and device position within the nasal cavity can significantly alter drug distribution and, consequently, absorption efficiency (Nguyen and Duong, 2025a). Therefore, nasal drug delivery strategies should fully consider these individual differences and improve deposition efficiency and reproducibility through optimized device design, controlled spray parameters, and personalized administration techniques.

Once individual differences are addressed, safety should be the primary consideration when designing intranasal formulations. Drug design should account not only for efficacy and bioavailability but also for local side effects, systemic effects, and CNS toxicity. Drugs delivered intranasally first contact the nasal mucosa, which serves as a barrier against harmful and exogenous substances. Nasal mucosal cilia play dual roles in clearing harmful substances and assisting drug delivery (Gizurarson, 2015; Zhang et al., 2023). The nasal microenvironment is also crucial for maintaining normal function. Therefore, drug design should consider potential damage to the nasal mucosa, cilia, and microenvironment from long-term administration. This consideration extends beyond the drug itself to include carriers and excipients. For intranasal delivery to treat CNS diseases, neurotoxicity must also be evaluated, especially when nanoparticles are used as carriers to increase bioavailability. Long-term, systematic assessment of the neurotoxicity of nanoparticle carriers is necessary. However, studies, such as intranasal baicalin liposome treatment for cerebral ischemia‒reperfusion injury in rats, have shown greater efficacy than intravenous administration without detectable nasal mucosal damage (Xiang et al., 2020). *In vitro* studies have shown that curcumin nanoemulsions have no cytotoxicity or ciliary toxicity; these are short-term studies (Sood et al., 2014). Research on whether long-term nasal administration induces local, systemic, or neurotoxic effects remains limited.

Recently, nanoparticle-based intranasal drug delivery to increase absorption and bioavailability has become mainstream, but nanoparticle-associated toxicity cannot be ignored. Buzea et al. (2007) summarized nanoparticle toxicity, including respiratory diseases (e.g., asthma, bronchitis, and lung cancer), neurodegenerative diseases (e.g., PD and AD), gastrointestinal diseases, and cardiovascular diseases (e.g., atherosclerosis and thrombosis). Nanoparticle toxicity depends not only on their “nano” properties but also on chemical composition, particle size, shape, aggregation state, exposure route, and individual differences. Studies have confirmed that intranasal nanoparticle carriers may cause mild nasal mucosal toxicity. Future research should focus on nanoparticle clearance after CNS delivery and long-term CNS effects and systematically identify nanoparticle types, structures, shapes, and sizes that may induce neurotoxicity, which will help reduce neurotoxic risks and inform occupational and environmental risk assessments amid increasing airborne nanoparticle exposure.

Although intranasal drug delivery for CNS diseases has been preliminarily validated in animals, significant challenges remain for clinical translation. In drug development and approval, regulatory oversight should be strengthened, with attention to long-term toxicity to ensure that repeated administration does not produce uncontrolled adverse effects. Standardization of bioavailability and pharmacokinetics is needed, along with personalized nasal delivery devices. Before approval, strict adherence to phase III clinical trials is needed, alongside robust post-marketing pharmacovigilance to monitor adverse reactions and potential misuse and strengthen traceability. Ethical standards must be strictly followed throughout research and clinical application to ensure the safety, efficacy, and controllability of intranasal drug delivery in clinical practice.

## Limitations

This review has several limitations. First, at the study level, many of the included studies are based on animal models, which may not fully reflect the anatomical and physiological characteristics of humans, introducing a potential risk of bias when extrapolating findings to clinical settings. Additionally, individual studies often have small sample sizes, heterogeneous experimental designs, or lack appropriate controls, which may affect the robustness and reproducibility of the reported mechanisms. Second, at the review level, although a systematic search was performed, it is possible that some relevant literature, especially non-English publications or recent preprints, was not retrieved, leading to incomplete coverage. Moreover, the review may be affected by reporting bias in the original studies, as positive results are more likely to be published, and negative or null findings may be underrepresented. Finally, owing to the rapidly evolving nature of this field, some newly emerging techniques or findings may not be fully captured, limiting the comprehensiveness of the current review.

## Conclusion

This review systematically outlines the anatomical basis, immune mechanisms, and therapeutic strategies by which peripheral nasal inflammation contributes to CNS diseases. Recent research has demonstrated that the NBA is a multi-pathway, bidirectional communication system, with both its structural foundation and functional pathways confirmed by various visualization techniques. This finding highlights that the nasal cavity is not merely a sensory organ but also a direct window connecting the periphery and CNS, providing a theoretical basis for understanding the pathophysiological mechanisms through which peripheral inflammation affects the CNS and for developing “cross-system” targeted therapeutic strategies. Furthermore, this review summarizes the mechanisms by which nasal inflammation induces central neuroinflammation via NBA, including direct activation of microglia and astrocytes and stimulation of related inflammatory pathways, which directly affect the nervous system or indirectly influence the CNS by modulating central neuroregeneration. These insights offer new etiological perspectives and potential early intervention targets for mood disorders, migraines, PD, and AD. Finally, the review examines emerging strategies for brain-targeted therapy via intranasal drug delivery mediated by the NBA, highlighting the challenges of clinical translation and considerations for long-term safety. The goal is to promote the development of intranasal drug delivery for CNS diseases toward more rigorous, safe, and translatable approaches. To address existing challenges, future research should focus on constructing multidisciplinary technological platforms that integrate high-resolution dynamic imaging, computational modeling, and organoid models to achieve precise visualization and quantitative analysis of NBA pathways, thereby laying a theoretical foundation for standardizing drug delivery efficiency and bioavailability. On this basis, further studies can elucidate the dynamic regulatory networks of the NBA under physiological and pathological conditions and the synergistic interactions among its various pathways, providing new insights for cross-system disease research. Moreover, translational research should be actively promoted by designing animal models that better reflect human physiological and pathological states, conducting long-term longitudinal studies, systematically evaluating the efficiency and safety of different delivery strategies, and exploring individualized administration schemes to develop more efficient, low-toxicity, and precise cross-system targeted therapies.

## Data Availability

*Not applicable.*
